# Surviving environmental change: when increasing population size can increase extinction risk

**DOI:** 10.1098/rspb.2022.0439

**Published:** 2022-06-08

**Authors:** Mark M. Tanaka, Lindi M. Wahl

**Affiliations:** ^1^ University of New South Wales, Sydney, NSW 2052, Australia; ^2^ Western University, London, Ontario, Canada, N6A 5B7

**Keywords:** evolutionary rescue, standing variation, extinction, mathematical model

## Abstract

Populations threatened by an abrupt environmental change—due to rapid climate change, pathogens or invasive competitors—may survive if they possess or generate genetic combinations adapted to the novel, challenging condition. If these genotypes are initially rare or non-existent, the emergence of lineages that allow a declining population to survive is known as ‘evolutionary rescue’. By contrast, the genotypes required for survival could, by chance, be common before the environmental change. Here, considering both of these possibilities, we find that the risk of extinction can be lower in very small or very large populations, but peaks at intermediate population sizes. This pattern occurs when the survival genotype has a small deleterious effect before the environmental change. Since mildly deleterious mutations constitute a large fraction of empirically measured fitness effects, we suggest that this unexpected result—an intermediate size that puts a population at a greater risk of extinction—may not be unusual in the face of environmental change.

## 1. Introduction

Populations that face a sudden and adverse change in their environment—for example through climate change, the emergence of novel pathogens or the arrival of invasive competitors—may be able to survive if some members of the population carry an allele, or combination of alleles, that allows survival in the novel, challenging condition. If the required allele is rare (or completely absent) before the environmental change, survival can occur via ‘evolutionary rescue’ [1–3], in which the pace of adaptive change outstrips population decline.

The simplest models of an environmental challenge assume that the change is instantaneous (but see [4]); after this change, the fitness of individuals that do not carry the survival allele is reduced, and such lineages begin to decline. If the population initially lacks the survival allele, evolutionary rescue can occur via de novo mutation, while the population is potentially en route to extinction. Survival can also occur via standing variation; this is also considered evolutionary rescue if the survival allele was rare before the environmental change [5,6].

For a given environmental challenge, however, the allele required to survive could in fact be common before the environment changes. For example, antibiotic resistance alleles may pre-exist at high frequencies in untreated patients, and more generally unselected alleles can reach high frequencies due to genetic drift. These situations are excluded, by definition, from evolutionary rescue, since the population is not in need of rescuing if the survival allele is common.

The relationship between population size and extinction is of long-standing interest in conservation biology [7–9]. In surviving an abrupt environmental change as described above, the survival probability is closely tied to the *number of copies* of the survival allele, rather than to the frequency of the allele in the population. This is because each copy has an independent chance of creating a lineage that will ultimately rescue the population, irrespective of the size of the wild-type population. If the survival allele is maintained in standing variation at a given frequency, this implies that a larger population would have a higher chance of survival because it carries more copies of the allele. Similarly, for a given mutation rate, a large population will create more copies of the required allele by de novo mutation before extinction. Finally, because extinction occurs at an absolute threshold of zero individuals, larger populations allow more time for rescue to occur before extinction. For these reasons, it seems reasonable that the probability of survival will increase with population size; larger populations appear to be safer from extinction.

Indeed, as we will illustrate below, the probability of *evolutionary rescue* increases with population size; larger populations have an advantage when the required allele is initially absent or rare. But does this hold more generally? If a particular allele is required to survive an environmental change, what is the overall probability that the population survives, allowing for the possibility that the allele could be at any frequency (absent, rare or common) when the population faces the novel challenge?

To address this question rigorously, further assumptions are required regarding the selective effect of the survival allele in the environment before the change, and the population sizes of interest. While we delineate these assumptions mathematically in the sections to follow, the essence of our approach is that (1) genetic drift may be non-negligible in the small populations that will be at risk of extinction in relevant scenarios and (2) it is very common for mutations to have mildly deleterious effects—that is, effects that are not completely neutral but are far from lethal. We demonstrate that under these conditions, survival can be favoured in smaller populations, because genetic drift maintains mildly deleterious alleles at substantial frequencies when the population size is small. Thus the survival probability can in fact decrease with increasing population size. This counterintuitive result may have broad implications for conservation, antimicrobial resistance and pathogen escape from immune or vaccine pressure [1,2].

## 2. Methods

We combine a well-studied model of evolutionary rescue [5,6] with Wright’s classic model of the balance among genetic drift, selection and mutation [10] to describe the survival probability as a function of population size. For simplicity, we consider a haploid population of size *N* that begins to decline at rate *δ* per generation after an instantaneous change in the environment; that is, the expected number of offspring per wild-type individual (absolute fitness) is initially one, and instantaneously becomes 1 − *δ* in the novel environment. We assume that an allele (or set of alleles that can be grouped) at a locus confers the ability to withstand this change in environment. Specifically, the survival allele has a fitness of 1 − *δ* + *s*_*b*_ after the change, where *s*_*b*_ > *δ*. Before the change, we assume that the survival allele bears a fitness cost *s*_*d*_. Mutation is reversible; mutation from wild-type to the survival allele and *vice versa* occurs at rate *μ*. This model is equivalent to the model proposed and analysed by Orr and Unckless for evolutionary rescue, with the additional simplification that the forward and back mutation rates are equal [5,6]; this assumption could easily be relaxed, as we discuss in later sections.

Orr & Unckless [6] derived a compact expression for the survival probability, including the possibility that rescue might occur either through de novo mutation, or through standing variation. This approximation can be understood as

2.1 $$P\{\mathrm{survival}|p\}=1-e^{-\pi (s_{b}-\delta )(A_{\mathrm{st}}\;+\;A_{\mathrm{dn}})}.$$

Here, *π*(*s*) denotes the establishment probability of a single allele with selective effect *s*, while *A*_st_ is the number of copies of the survival allele in standing variation at the time of the environmental change and *A*_dn_ is the number of copies that will be created by de novo mutation during the population decline. The survival probability given here depends on *p*, the frequency of the survival allele in the population at the moment when the environment changes. From this definition of *p*, it is clear that *A*_st_ = *pN*. The total number of new mutations that occur while the population declines, *A*_dn_, is given by the sum of a geometric series describing the expected number of mutations in each generation of this decline toward extinction. Orr and Unckless assume that *p* ≪ 1; that is, the population is nearly fixed for the wild-type allele at the time of the change. In this case, *A*_dn_ ≈ *Nμ*/*δ* [6].

In the sections to follow, we will consider population survival across a range of population sizes, including small populations experiencing strong genetic drift; thus we will treat cases in which *p* may be large. We therefore generalize the expression for *A*_dn_ to include the fact that the rescue allele may already be present, yielding *A*_dn_ = (1 − *p*)*Nμ*/*δ*. We note that this generalization will have little effect on the overall survival probability, since when *p* is large, survival is already very likely through standing variation. However, it may influence the balance between de novo and standing variation as described in §3.

We will also treat some cases in which the survival allele has a relatively large effect, and thus we replace *π*(*s*) ≈ 2*s* (as used in [6]) with the larger root of the equation *π*(*s*) = 1 − e^−(1+*s*)*π*(*s*)^ [11]; this approximation assumes a branching process with Poisson-distributed offspring numbers, and has also been shown to be applicable to a declining population [12], but does not assume that *s* is small. These generalizations yield the survival probability for a given initial frequency *p*:

2.2 $$P\{\mathrm{survival}|p\}=1-e^{-N\pi (s_{b}-\delta )(p+(1-p)\mu /\delta )}.$$

The frequency of the survival allele at the time of the change, *p*, will not generally be known. If we let *ϕ*(*p*) describe the probability density function for *p*, then as described previously [5], to compute the overall probability of survival, we need to average equation (2.2) over all possible allele frequencies

2.3 $$P_{\text{survival}}=\int_{\;p=0}^{1}\phi (p)P\{\mathrm{survival}|p\}\;dp,$$

2.4 $$\;=1-\int_{0}^{1}\phi (p)\exp\left(-N\pi (s_{r})\left(\;p\left(1-\frac{\mu }{\delta }\right)+\frac{\mu }{\delta }\right)\right)\;dp,$$

where we take *s*_*r*_ = *s*_*b*_ − *δ* to simplify the notation going forward.

### (a) Stationary distribution of the survival allele

To predict survival probabilities, all that remains is to describe the probability density function *ϕ*(*p*), the stationary distribution of the survival allele, at population size *N*, before the environmental change. When the forces of drift, mutation and selection are all non-negligible, one approach is to assume that the stationary distribution for the survival allele frequency is given by Wright’s equation [10]

2.5 $$\phi (p)=C\;e^{-2Ns_{d}p}{\left(p(1-p)\right)}^{2N\mu -1},$$

where *C* is a constant defined such that the distribution *ϕ*(*p*) integrates to unity

**Figure 1.**
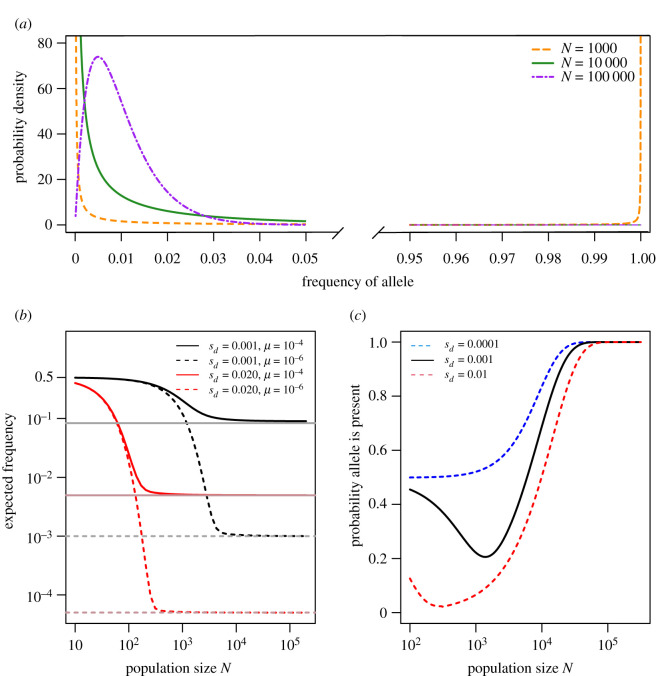
Wright’s steady-state distribution. (*a*) The stationary distribution of a survival allele (equation 2.5) with selective disadvantage *s*_*d*_ = 0.001, under symmetric mutation, in populations of size *N* as indicated. In small populations, the distribution is bimodal with a substantial probability that the population is fixed or nearly fixed for the survival allele. (*b*) The mean frequency of an allele under Wright’s distribution, with symmetric mutation at the rates indicated. The mean is computed numerically using equation (2.7). At low population sizes, drift dominates, so the mean frequency is near 0.5, which reflects the balance between two equal mutation rates. At high population sizes, the frequency is determined by the balance between mutation and selection; in this case $\bar{p}$ approaches the deterministic equilibrium approximated by $\hat{\;p}=\mu /(s+2\mu )$, shown here with faint horizontal lines. (*c*) The probability that at least one copy of the survival allele is present in the population at steady state (equation (2.8)), as a function of population size. Mutation is symmetric at rate *μ* = 10^−5^ in (*a*) and (*c*).

2.6 $$\frac{1}{C}=\int_{0}^{1}\;e^{-2Ns_{d}p}{\left(p(1-p)\right)}^{2N\mu -1}\;dp,$$

and we have assumed that forward and backward mutation rates are equal (figure 1*a*).

Equation (2.5) admits a number of simplifying cases, depending on the cost of the survival allele, *s*_*d*_, before the environment changes. If *s*_*d*_ is sufficiently large, the survival allele is rare (*p* ≪ 1) and back mutation can be ignored. In this case, *ϕ*(*p*) can be approximated by a gamma distribution [13]. By contrast, if *s*_*d*_ is sufficiently small, the survival allele is effectively neutral. In this case, *ϕ*(*p*) can be approximated by a beta distribution [14]. Orr & Unckless [5] consider these two cases in detail.

A limitation of Wright’s stationary distribution is that the expected time to approach this equilibrium can be very long, at a timescale given by the inverse of the mutation rate. Another approach is to assume that the survival allele appears, drifts transiently and is lost. Hermisson & Pennings [15] use a diffusion approximation to derive simple approximations for *ϕ*(*p*) in this situation, conditioned on the survival allele never reaching fixation and assuming that its fitness effect is either neutral or deleterious. Again, the survival allele frequency is assumed to be small (*p* ≪ 1).

As outlined in §1, we sought to generalize these approaches for two reasons. First, we reasoned that populations at risk of extinction would often be small. We wanted to derive the extinction probability for populations on the order of hundreds or a few thousand individuals [9,16,17]. In populations of that size, genetic drift can be powerful, and thus the frequency of a deleterious allele may not always be small. Second, empirically measured distributions of mutational effects often show a large fraction of mildly deleterious mutations (for review, see [18]). We therefore assumed that this intermediate case, in which the survival allele is neither strongly deleterious nor effectively neutral, might in fact be a common scenario. Orr & Unckless [5] treat this case briefly, but do not explore the effects of population size on the survival probability.

We thus wished to retain the full stationary distribution as derived by Wright [10]. To gain an intuitive understanding of the results to follow, it is helpful to first understand $\bar{p}$, the mean frequency of the survival allele prior to the environmental change. This is given by

$$\bar{\;p}=\int_{0}^{1}\phi (p)\;p\;dp.$$

Kimura *et al.* [19] derive $\bar{p}$ in terms of the _1_*F*_1_ confluent hypergeometric function [20]. When the forward and back mutation rates are equal, the mean frequency of the allele at steady state is

2.7 $$\bar{\;p}=\frac{1}{2}\frac{{\;}_{1}F_{1}(\alpha +1,2\alpha +1,-\gamma )}{{\;}_{1}F_{1}(\alpha ,2\alpha ,-\gamma )},$$

where *α* = 2*Nμ* and *γ* = 2*Ns*_*d*_.

Figure 1*b* shows $\bar{p}$ as a function of *N*. For large *N*, $\bar{p}$ approaches the deterministic mutation-selection balance approximated by $\hat{\;p}=\mu /(s_{d}+2\mu )$, which differs from the classic expression *μ*/*s*_*d*_ because we do not assume that back mutation is negligible.

When *N* is small, however, $\bar{p}$ approaches 0.5 irrespective of *μ* and *s*_*d*_. This is because drift is increasingly strong, overwhelming selection against the deleterious allele. Thus, when mutation is symmetric, the average frequency of the survival allele before the environment changes can be close to 1/2 in small populations. More generally, if *u* is the mutation rate to the survival allele and *v* is the back mutation rate, $\bar{p}$ will approach *u*/(*u* + *v*) at small *N*. We note, however, that this result does not imply that many small populations have a survival allele frequency close to 0.5. Rather, small populations tend to be fixed for either the survival or the wild-type allele, as shown in figure 1*a*. Finally, we note that since Wright’s distribution is a diffusion approximation of the Wright–Fisher model, it is most accurate at large *N*. However, comparisons against full Wright–Fisher simulations show that the approximation performs very well even at *N* = 10, 100 and 200 (electronic supplementary material, figures S1 and S2).

We can also use Wright’s stationary distribution to estimate the probability that at least one copy of the survival allele exists, in standing variation, when the environment changes. This probability is simply given by

2.8 $$P\{\mathrm{at}\;\mathrm{least}\;1\;\mathrm{copy}\}=\int_{1/N}^{1}\phi (p)\;dp.$$

When the survival allele is mildly deleterious, this probability drops substantially at intermediate population sizes (figure 1*c*), as selection against the deleterious allele begins to dominate drift. As the population size increases, however, the probability that the survival allele exists approaches unity. This non-monotonic behaviour underpins the results presented in the next section.

### (b) Survival probability as a function of population size

Substituting Wright’s stationary distribution, equation (2.5), into equation (2.4), the survival probability can be written as

2.9 $$P_{\mathrm{survival}}=1-C\;e^{-N\pi (s_{r})\mu /\delta }\int_{0}^{1}{\left(p(1-p)\right)}^{2N\mu -1}\;e^{-2N\tilde{s}p}\;dp$$

2.10 $$\;=1-e^{-N\pi (s_{r})\mu /\delta }\frac{\int_{0}^{1}{\left(p(1-p)\right)}^{2N\mu -1}\;e^{-2N\tilde{s}p}\;dp}{\int_{0}^{1}{\left(p(1-p)\right)}^{2N\mu -1}\;e^{-2Ns_{d}p}\;dp},$$

where we use $\tilde{s}$ to denote *s*_*d*_ + (1 − *μ*/*δ*)*π*(*s*_*r*_)/2, and we use *C* as defined in equation (2.6).

Note that the integral representation of the confluent hypergeometric function of the first kind is

$${\;}_{1}F_{1}(\alpha ,\beta ,z)=\frac{\Gamma (\beta )}{\Gamma (\alpha )\Gamma (\beta -\alpha )}\int_{0}^{1}p^{\alpha -1}{\left(1-p\right)}^{\beta -\alpha -1}\;e^{zp}\;dp$$

[20]. Therefore, letting *β* = 2*α*, we have

2.11 $$\int_{0}^{1}{\left(p(1-p)\right)}^{\alpha -1}\;e^{zp}\;dp=\frac{{\;}_{1}F_{1}(\alpha ,2\alpha ,z)\Gamma^{2}(\alpha )}{\Gamma (2\alpha )}.$$

Substituting this expression into the numerator and denominator of equation (2.10), we find

2.12 $$P_{\text{survival}}=1-e^{-N\pi (s_{r})\mu /\delta }\;\frac{{\;}_{1}F_{1}(2N\mu ,4N\mu ,-2N\tilde{s})}{{\;}_{1}F_{1}(2N\mu ,4N\mu ,-2Ns_{d})},$$

(see for comparison eqn (9) in [5]). As demonstrated in §3 to follow, this expression exhibits a number of interesting behaviours as *N* varies, and is only monotonic in *N* in some limiting cases.

### (c) Simulations

The analytical predictions described above were confirmed in stochastic simulations of haploid populations of initial size *N*. The simulated process starts at the moment of the environmental change, and is followed until either the survival allele is established (see below) or the population goes extinct. Recall that at the moment of the environmental change, the survival allele frequency, *p*, should be distributed according to Wright’s distribution, equation (2.5). The initial condition for *p* was therefore drawn from Wright’s distribution by integrating equation (2.5) in intervals with boundaries (0, 0.5/*N*, 1.5/*N*, …, (*N* − 1.5)/*N*, (*N* − 0.5)/*N*, 1), where the two edge bins have width 0.5/*N* while the others have width 1/*N*. Thus, apart from the two edge bins, each bin is centred at allele frequency *j*/*N* for *j* = 1, 2, …, *N* − 1. Because the density function is very steep near the boundaries at low population sizes, we integrated using a numerical approach devised by Kimura [19] for smaller *N* values. These *N* + 1 integrated probability masses were used to draw a random frequency *p* from (0, 1/*N*, …, (*N* − 1)/*N*, 1), which we used to initialize the simulation. We also note that this discretization of equation (2.5) is the simplest, but least accurate, of methods discussed by Ewens ([21]; see pp. 177–178). We also implemented the discretization believed to be most accurate (Ewens [21], equation (5.79)) but this yielded indistinguishable results for survival probabilities.

Using *L*_*i*_ and *M*_*i*_ to denote the number of individuals carrying the wild-type and survival alleles, respectively, in generation *i*, we first set the initial number of copies of the survival allele *M*_0_ to *pN* and the number of wild-type *L*_0_ to (1 − *p*)*N*, each rounded to the nearest integer. In each generation *i*, the simulation algorithm determines the total number of offspring, then accounts for mutation. First, the number of wild-type offspring that will form generation *i* + 1 is drawn from a Poisson distribution with mean (1 − *δ*)*L*_*i*_, while the number of offspring in the survival lineage is drawn from a Poisson distribution with mean (1 − *δ* + *s*_*b*_)*M*_*i*_. Mutations at the survival locus occur independently in each offspring individual with probability *μ*; the number of wild-type alleles in generation *i* + 1 is then given by the sum of the wild-type offspring in which mutation did not occur, and survival-allele offspring in which mutation did occur. The number of survival alleles in generation *i* + 1 is computed analogously, since mutation is symmetric.

The process is stopped when either the population goes extinct or the survival lineage reaches a fixed threshold size, indicating that survival has succeeded. We used a threshold of 2000 individuals but our results are insensitive to this number, as long as it is large enough that the survival lineage is unlikely to go extinct after reaching the threshold.

Both the mathematical and simulation approaches above assume that lineages carrying the survival allele reproduce independently, a common simplifying assumption in evolutionary rescue (but see for example [22,23]). Since this assumption can fail when the survival allele is not initially rare, we also simulated populations in which population size is regulated according to the Ricker model [24]. In these simulations, each wild-type individual has offspring with mean number given by exp (*r*[1 − *N*_*t*_/*K*_*t*_]), where *r* > 0 is the intrinsic growth rate, *N*_*t*_ is the total population size *t* generations after the environmental change and *K*_*t*_ is the carrying capacity in generation *t*, which declines according to *K*_*t*_ = *K*_0_(1 − *δ*)^*t*^. By contrast, individuals carrying the survival allele have mean offspring number exp (*r*[1 − *N*_*t*_/*K*_0_]) in generation *t*; their carrying capacity is unaffected by the environmental change. Populations are initialized at the Wright stationary distribution with a population size *N*_0_ equal to the carrying capacity *K*_0_. Note that in contrast with our analytical work, in this approach the relative fitness of the survival allele, analogous to *s*_*b*_, depends on the population size and varies over time.

## 3. Results

**Figure 2.**
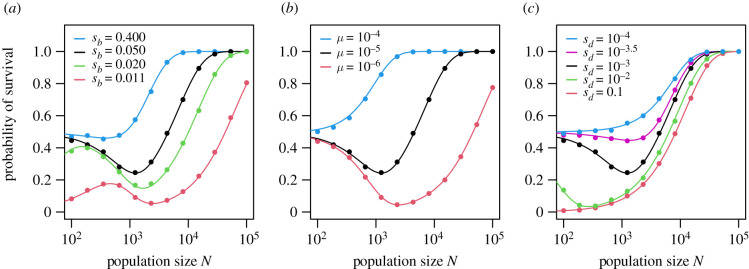
The probability of survival as a function of population size *N* before the change in environment. The curves show these probabilities calculated using equation (2.12). The points show probabilities estimated from 5000 simulations per point; these simulations are described in §2. We show the effect of varying *s*_*b*_ in (*a*), varying *μ* in (*b*) and varying *s*_*d*_ in (*c*). The default parameters are: *s*_*b*_ = 0.05, *δ* = 0.01, *s*_*d*_ = 0.001, *μ* = 10^−5^. In electronic supplementary material, figure S3, we show how the severity of the environmental change, *δ*, affects the survival probability.

Figure 2 shows the probability of survival as a function of population size, across a wide parameter range. In (*a*–*c*)—as we vary *s*_*b*_, *μ* or *s*_*d*_—we see regions in which the survival probability declines, often substantially, as the population size increases. While our analytical approach (solid lines) assumes that the allele frequency is continuous even at small *N*, these predictions are very closely confirmed by simulation (plotted points). This pattern persists when we relax the assumption that survival lineages act independently (electronic supplementary material, figure S4).

This dramatic non-monotonicity of survival with *N* disappears in several asymptotic cases. If either the benefit of the survival allele or the mutation rate is large (figure 2*a*,*b*, blue lines), survival grows monotonically. In figure 2*c*, taking *N*_neut_ = 1/*s*_*d*_ as the threshold for neutrality, we see that when *s*_*d*_ = 10^−4^, the survival allele is effectively neutral across the range of *N* plotted (up to 10^4^). In this case, survival simply grows with population size. By contrast, when *s*_*d*_ = 0.1 the allele is deleterious for *N* > 10, and again survival is nearly monotonic; these are the two cases studied in most detail by Orr & Unckless [5]. A local minimum in survival probability appears when *N*_neut_ is neither so small that survival is unlikely at *N*_neut_, nor so large that survival is nearly certain at this population size.

We also note that when *s*_*b*_ is small (*a*), very small populations have a high probability of going extinct, even when initially fixed for the survival allele. As a numerical example, if *s*_*b*_ − *δ* = 0.001 as shown here (red line), a single lineage carrying the survival allele will die out with probability *x* ≈ 0.998, and thus a population of 500 that is initially fixed for the survival allele will go extinct *x*^500^ ≈ 37% of the time. Since smaller populations are even more vulnerable to this demographic extinction, the survival probability increases with *N* when both *s*_*b*_ and *N* are small. For the population sizes investigated here, this effect disappears when the population size is stabilized by density-dependence in the Ricker model (electronic supplementary material, figure S4).

### (a) De novo versus standing variation

Another way to visualize this phenomenon is to examine the contributions of standing variation and de novo mutation to survival. To do this, we compute the probability of survival via standing variation as

3.1 $$P_{\mathrm{stand}}={\int_{0}^{1}\;P\{\mathrm{survival}|p\}|}_{\mu =0}\phi (p)\;dp.$$

Note that the condition *μ* = 0 is applied only to *P*{survival|*p*}, but not to *ϕ*(*p*). This expression thus gives the survival probability through standing variation only, as if de novo mutation were turned off at the moment of the environmental change. Similarly, we compute

3.2 $$P_{\mathrm{de}\;\mathrm{novo}}=P\{\mathrm{survival}|p=0\},$$

which gives the probability of survival given that there was no standing variation at the moment of the environmental change. The overall probability of survival can then be written as

3.3 $$P_{\mathrm{total}}=P_{\mathrm{stand}}+(1-P_{\mathrm{stand}})P_{\mathrm{de}\;\mathrm{novo}}.$$

**Figure 3.**
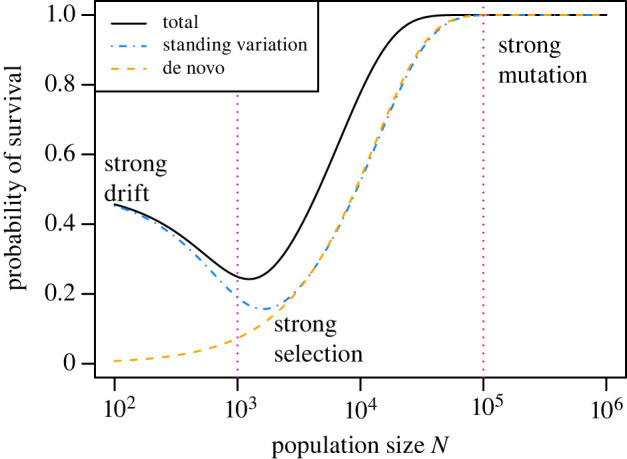
Components of the survival probability. Here, the blue curve gives the probability of survival via standing variation, assuming no new mutations occur after the environment changes (equation (3.1)) and the orange curve gives the probability of survival via de novo mutation only (equation (3.2)). The pink dotted vertical lines indicate *N*_neut_ = 1/*s*_*d*_ (left line) and *N*_mut_ = 1/*μ* (right line). Text labels indicate effects of population genetic forces on the survival probability. In the *strong drift* regime, the population size is small enough for drift to dominate so that the survival allele is at a mean frequency of around 0.5, and the absolute number of copies of the allele is large enough for survival to occur reliably. In the *strong selection* regime, purifying selection against the survival allele is strong since *s*_*d*_ *N* > 1 so that the allele is at a suppressed frequency before the environmental change, thus reducing the probability of survival. In the *strong mutation* regime, the mutation rate is so high that a successful survival allele is likely to exist in standing variation before the environmental change and also likely to appear by de novo mutation after the change. Parameters: *s*_*b*_ = 0.05, *δ* = 0.01, *s*_*d*_ = 0.001 and *μ* = 10^−5^. (Online version in colour.)

These components of survival are shown in figure 3. For comparison, we also plot *N*_neut_ = 1/*s*_*d*_ (left vertical line) and *N*_mut_ = 1/*μ* (right vertical line). *N*_mut_ indicates a simple but conservative threshold population size at which survival is nearly certain, since on average a new copy of the survival allele would be produced in every generation. In this figure, we can clearly see that selection due to the fitness cost *s*_*d*_ increases with population size as the population approaches *N*_neut_, reducing *P*_stand_. While *P*_de novo_ increases with *N*, the rate of increase is slow when *N* ≪ 1/*μ*. Survival declines with population size when the increase in *P*_de__novo_ is overwhelmed by the reduction in *P*_stand_ as the population size increases.

We have used the full distribution of allele frequencies in order to study the probability of a population surviving an abrupt environmental change. This survival would be defined as evolutionary rescue when the initial frequency of the survival allele is rare, but how rare? One possibility is to restrict ‘evolutionary rescue’ to cases in which the population fitness, at the time of the environmental change, is less than unity, which corresponds here to *p* < *δ*/*s*_*b*_. When considering evolutionary rescue scenarios defined in this way, we find that the standing variation component and thus the overall survival probability increase monotonically with increasing population size (electronic supplementary material, figure S5). In fact, this monotonic increase holds if we exclude only cases in which the survival allele is fixed, through genetic drift, in the population before the environmental change. We suggest, however, that use of the full distribution of allele frequencies is more general and ecologically pertinent; for a given change in the environment and a particular allele necessary for survival (eg. antibiotic resistance), situations in which the allele is fixed before the change make an important and non-negligible contribution to the survival probability of the population as a whole.

### (b) What population size maximizes the extinction probability?

The local minima in figure 2 highlight populations that are more vulnerable to extinction, counterintuitively, than they would be if they were smaller; in some parameter regimes, these intermediate population sizes are at greater risk of extinction than even very small populations (e.g. when mutation is rare; see red line in figure 2*b*). Given the parameters of a survival scenario, can we predict whether such a region of unexpected vulnerability exists? In other words, is there a ‘dip’ in the survival probability as the population size increases, and if so, where is it centred?

Defining *A* = *π*(*s*_*r*_)*μ*/*δ*, we can re-write equation (2.10) as

3.4 $$P_{\text{survival}}=1-e^{-AN}\frac{\int_{0}^{1}{\left(p(1-p)\right)}^{2N\mu -1}\;e^{-2N\tilde{s}p}\;dp}{\int_{0}^{1}{\left(p(1-p)\right)}^{2N\mu -1}\;e^{-2Ns_{d}p}\;dp}$$

3.5 $$\;=1-e^{-AN}\frac{G(N,\tilde{s})}{G(N,s_{d})},$$

where

$$G(N,s)=\int_{0}^{1}\frac{{\left[{\left(p(1-p)\right)}^{2\mu }\;e^{-2sp}\right]}^{N}}{p(1-p)}\;dp.$$

Denoting the derivative ∂*G*/∂*N* = *G*′(*N*, *s*) and setting the derivative *dP*_survival_/*dN* to zero, we find that critical population sizes must satisfy

3.6 $$\begin{array}{r} G^{\prime }(N,\tilde{s})G(N,s_{d})-G(N,\tilde{s})G^{\prime }(N,s_{d})=AG(N,\tilde{s})G(N,s_{d}). \end{array}$$

In principle, we can solve equation (3.6) for values of *N* at which the probability of survival is either maximized or minimized. Since at large *N*, *P*_survival_ increases and saturates, the largest root of equation (3.6), when such a root exists, will yield a local minimum corresponding to a vulnerable intermediate population size. In practice, since equation (3.6) must be solved numerically, numerical optimization can also be used to directly find the local minima of equation (2.10). (We cannot exclude the possibility of more than one local minimum, but did not observe more than one in any of the parameter regimes we tested.)

A local minimum in the survival probability occurs due to the balance of two factors. As the population size grows, selection against deleterious alleles grows more effective, reducing the frequency of the survival allele in standing variation. At a given mutation rate, however, the number of copies of the allele—a key predictor of survival—increases as the population size grows. The survival probability declines with population size when the force of selection against the survival allele increases faster than the mutation pressure creating new copies of the allele.

**Figure 4.**
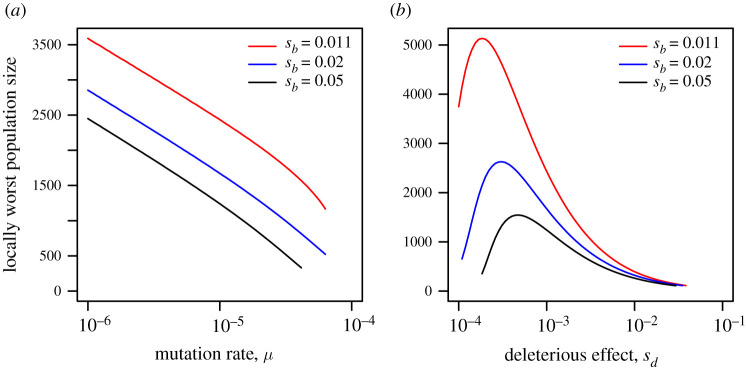
The population size at which the survival probability is (locally) minimized, as a function of *μ* ((*a*), *s*_*d*_ = 0.001) and of *s*_*d*_ ((*b*), *μ* = 10^−5^) for different values of *s*_*b*_. These were found by numerical minimization of equation (2.12) as a function of *N* to estimate the rightmost local minimum. These local minima reflect population sizes that are more vulnerable to extinction than smaller populations facing an equivalent survival challenge. Note that the local minimum exists over a wide range of parameter space, but does not always exist. In (*a*,*b*), *δ* = 0.01.

The population size that yields a local minimum in survival probability is plotted in figure 4. We see that under lower mutation pressure, larger populations are most vulnerable (*a*), since a larger population is then required to create sufficient copies of the survival allele. Similarly, more copies of the survival allele are required to escape loss by drift when the benefit of the survival allele, after the change, is small; in this case again the most vulnerable population size is increased. In (*b*), curves are truncated when a local minimum does not exist; we see as before that if the effect of the survival allele before the change is either strongly deleterious or nearly neutral, the minimum disappears (as seen in figure 2*c*). Note that as *s*_*d*_ increases, the survival allele becomes effectively neutral at smaller population sizes. Thus the local minimum occurs in smaller populations, as seen in the right in (*b*), as drift overwhelms purifying selection. Although we observe the opposite trend when *s*_*d*_ is very small (left side of (*b*)), survival curves in this regime are relatively flat (figure 2*c*) so the minimum is less relevant.

## 4. Discussion

We demonstrate that for small populations, the chance of surviving a sudden, adverse change in the environment can decrease with population size. This counterintuitive effect occurs when the allele required for survival is effectively neutral and thus maintained by drift in small populations, but is efficiently purged by selection in larger populations.

More specifically, for population sizes below the neutrality threshold, *N*_neut_ = 1/*s*_*d*_, a survival allele with cost *s*_*d*_ is ‘nearly neutral’. We can similarly consider the threshold *N*_mut_ = 1/*μ* as the population size at which survival is assured, since the survival allele would be created in every generation on average. The risk of extinction can increase with population size when *N*_neut_ is substantially less than *N*_mut_, or in other words whenever *s*_*d*_ is substantially larger than *μ*. Of course, if *s*_*d*_ is so large that the survival allele is deleterious at all relevant population sizes, this effect disappears. While our numerical exploration indicates a wide parameter regime over which survival is non-monotonic in population size, a precise analytical understanding of the boundaries defining this regime presents a challenging question for future work.

We illustrate results for population sizes as small as several hundred, but clearly these very small populations would be at risk of extinction, even in the absence of an environmental change, through processes such as mutational meltdown (the accumulation of deleterious mutations) [17,25] and other ‘extinction vortices’ [8]. Thus it seems unlikely that such small populations would persist for sufficient time (approximated by the inverse of the mutation rate) to reach Wright’s stationary distribution. Our results demonstrate, however, that survival probability can decrease with population size for populations of between 10^3^ and 10^4^ individuals (figures 2 and 4).

Why has this effect not been observed before? Orr & Unckless [5] use separate, analytically tractable approaches for neutral and strongly deleterious alleles, which they examine in detail. As seen in figure 2*c*, in both of these asymptotic cases (blue and red curves), the survival probability grows monotonically with population size. Orr and Unckless also use Wright’s stationary distribution to compute the rescue probability, via standing variation, for mildly deleterious alleles (see fig. 5, [5]). Although focusing on evolutionary rescue, results in that paper did not explicitly exclude cases in which the rescue allele is initially common, but also did not explicitly investigate changes in population size. Our results essentially extend this approach, allowing us to simultaneously address neutral, mildly deleterious and fully deleterious cases as we vary *N* over several orders of magnitude. While it is true that results for mildly deleterious alleles fall between predictions for neutral or strongly deleterious alleles (figure 2*c*), these two well-studied extremes lack the complex and surprising behaviour that dominates the intervening parameter space.

Our work makes a simple prediction that could be experimentally testable: does the probability of survival, in the face of an abrupt environmental change, drop as the population size increases toward *N*_neut_? Testing this hypothesis directly would require the survival allele frequency to reach equilibrium (Wright’s stationary distribution) before the environmental change, a condition that may be difficult to achieve in many protocols. In the future, the analytical approach we develop here could be extended to non-equilibrium settings. Deriving the transient behaviour of the survival allele (see [15]) would allow quantitative predictions for survival probabilities when the survival allele has only equilibrated for an experimentally feasible timescale before the environmental challenge. In particular, studying transient behaviour with an initially heterogeneous population (*p* ≠ 0, 1) might considerably ameliorate the 1/*μ* equilibration timescale.

Our model assumes a biallelic locus with symmetric mutation rates between the survival and wild-type alleles. More generally, these two alleles could represent two sets of genetic states that do and do not confer the survival advantage, respectively. In this case, the mutation rate to the survival state, *μ*, would not generally equal the mutation rate away from the survival state, *ν*; these rates would instead depend on the number of (accessible and viable) genetic states in each category. The expected frequency of the survival allele would then approach ≈ *μ*/(*μ* + *ν*) in very small populations, rather than ≈ 0.5 in the symmetric case. These considerations imply that whenever *μ* < *ν*, the reduction in survival probability with increasing population size would be less dramatic than the cases we have illustrated here.

Nonetheless, the understanding that population survival, at least in the face of an abrupt change, does not always increase with population size could have important implications. For situations in which survival is not the desired outcome (e.g. antibiotic resistance), an intermediate population size in the pathogen, before the antibiotic is applied, could minimize the probability that a mildly deleterious resistance allele is present; in this situation, it is feasible that both the cost and benefit of the resistance allele might be known, allowing a quantitative prediction of the most vulnerable pathogen population size. For pathogens that undergo repeated transmission bottlenecks, further investigation is warranted; however, the bottleneck size itself is likely the relevant population size for standing variation. If so, our results suggest that either very small or very large transmission bottlenecks favour resistance. After a very small bottleneck, the resistance allele may be fixed in the population by chance before therapy; for very large bottlenecks, the de novo emergence of resistance is likely. At intermediate bottleneck sizes, the resistance allele may be present but has a high chance of being outcompeted by the sensitive strain before therapy begins.

When considering endangered populations, our results suggest that several small subpopulations, in which slightly deleterious alleles could drift to high frequency, might be more likely to survive a sudden environmental change than one large, well-mixed population. Such a scenario is reminiscent of Wright’s shifting balance theory [26,27], though our analysis focuses narrowly on the probability of extinction or adaptation after an environmental change via a specific survival allele, rather than on adaptation on complex landscapes. Previous theoretical work on rescue in a structured population has identified a counterintuitive non-monotonic response of the rescue probability to the migration rate [23,28] and the rate of environmental degradation [23]. Future work could characterize the effect of population size in a structured population, noting that small subpopulations would be subject to the accumulation of other deleterious genes that would further endanger them [17,25]. More speculatively, if we consider the mutations required for a zoonotic pathogen to transmit efficiently in humans, these rare alleles may be deleterious in the animal reservoir, and thus the chance of zoonotic escape may also be non-monotonic as the size of the reservoir increases.

## Data Availability

All of the simulation code used in this study is freely available at https://github.com/m-tanaka-unsw/nonmon-survival. There are no data to be archived. Electronic supplementary material is available online [29].
